# Pseudoangiomatous stromal hyperplasia causing bilateral galactomastia with short-term recurrence after surgery: a case report

**DOI:** 10.1177/03000605241293684

**Published:** 2024-11-07

**Authors:** Zhouyang Lian, Xianzan Chen, Chunling Liu

**Affiliations:** Department of Radiology, Guangdong Provincial People’s Hospital, Guangdong Academy of Medical Sciences, China

**Keywords:** Diffuse pseudoangiomatous stromal hyperplasia, pseudoangiomatous stromal hyperplasia, breast, imaging, mastectomy, case report

## Abstract

Pseudoangiomatous stromal hyperplasia (PASH) is a rare, benign breast lesion characterized by collagen proliferation, often identified as an incidental microscopic finding. Clinically, it may present as a palpable, well-defined breast mass or, in rare instances, as a diffuse, bilateral process leading to significant breast enlargement. We herein report a case of extensive diffuse PASH with short-term recurrence following lesion resection at another hospital. Because of the severity of symptoms, the patient underwent bilateral mastectomy at our facility. This article reviews the clinical, radiologic, and pathologic features of PASH and emphasizes the importance of an accurate preoperative imaging diagnosis for guiding appropriate treatment.

## Introduction

Pseudoangiomatous stromal hyperplasia (PASH) was first described by Vuitch et al. in 1986.^1^ PASH is rarely the primary pathological finding and instead often coexists with other breast diseases, making it easy to overlook or misdiagnose.^2^ The term “pseudohemangioma” refers to slit-like, spindle cell-lined spaces that resemble small vessels in histopathology. Clinically, PASH can range from asymptomatic lesions detected during screening to palpable, symptomatic masses. When symptomatic, it most commonly presents as a palpable unilateral mass.^3,4^ In rare cases, rapidly growing, bilateral breast enlargement accompanied by skin thickening and erythema has been reported.^5–7^

The etiology and pathogenesis of PASH remain unclear. Given that PASH predominantly occurs in perimenopausal women and is infrequently seen in patients with gynecomastia or postmenopausal women receiving hormone therapy, it is considered hormone-dependent.^3,8,9^ Although PASH is not a risk factor for breast cancer or a precancerous condition, it is incidentally associated with benign or malignant breast lesions in up to 23% of instances.^8,9^

We herein describe a case involving a 53-year-old woman who presented with bilateral galactomastia and experienced short-term recurrence after surgery. This case is being reported to inform clinicians that in patients with diffuse PASH, mastectomy should be performed to prevent recurrence, and magnetic resonance imaging is very important for a correct preoperative diagnosis.

## Case report

**Figure 1.**
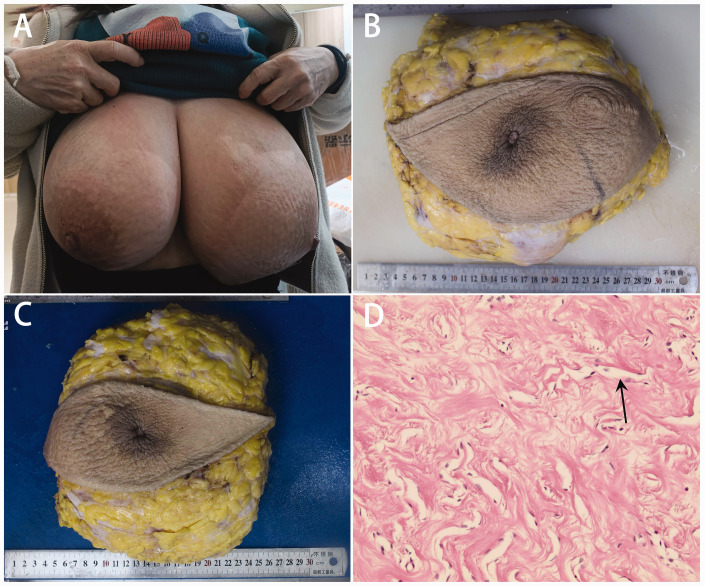
Bilateral galactomastia and histological examination in a patient with pseudoangiomatous stromal hyperplasia. (a) Bilateral galactomastia. After bilateral mastectomy, (b) the right breast specimen weighed 5275 g and (c) the left breast specimen weighed 6493 g. (d) Histological examination of the tissue revealed stromal hyperplasia and slit-like spaces (marked with arrows) lined by endothelial-like spindle cells.

A 53-year-old woman presented with bilateral breast pain and enlargement 2 years previously. She underwent bilateral breast lesion resection under general anesthesia at another hospital, and postoperative pathology confirmed a diagnosis of PASH. After the surgery, the patient reported progressive bilateral breast enlargement, with cyclical changes associated with menstruation (painful during menstruation and softening afterward), which impacted her daily life. Six months previously, she visited our hospital for further diagnosis and treatment because of movement limitations and respiratory difficulties. Physical examination revealed asymmetrical bilateral gigantomastia, with the left breast extending to the umbilical level and the right breast positioned approximately two finger breadths above the umbilical level (Figure 1(a)). Laboratory analysis revealed normal levels of estrogen and progesterone.

**Figure 2.**
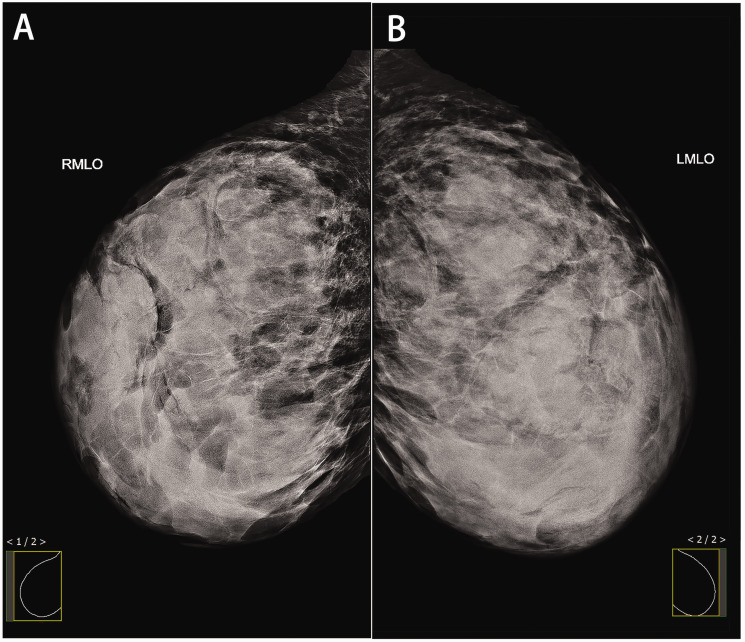
Mammography findings in a patient with pseudoangiomatous stromal hyperplasia. Mammography of the (a) right breast and (b) left breast showed diffuse enlargement without clear calcifications, and multiple masses appeared to be present.

**Figure 3.**
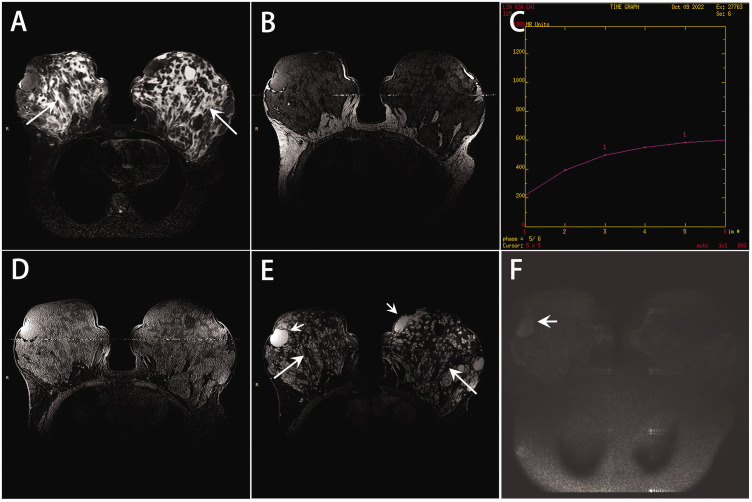
Magnetic resonance imaging findings of pseudoangiomatous stromal hyperplasia. (a) T2-weighted imaging showed high-signal slit-like spaces (long arrows). (b) T1-weighted imaging showed low-signal areas, and (d) T1-weighted fat suppression imaging also demonstrated low signals. (e) These slit-like spaces (long arrows) showed no enhancement, while multiple oval-shaped masses (short arrows) with clear boundaries were observed. (c) The dynamic enhancement curve was ascending. (f) Diffusion-weighted imaging revealed mild diffusion restriction within the enhanced masses.

Mammography indicated diffuse enlargement of both breasts without visible calcifications and appeared to show multiple masses (Figure 2). Magnetic resonance imaging revealed bilateral breast enlargement with multiple slit-like spaces (Figure 3) exhibiting low signals on T1-weighted images and high signals on T2-weighted images. These spaces showed no enhancement. Multiple oval-shaped masses with clear boundaries were observed within the lesions, with an ascending dynamic enhancement curve. Diffusion-weighted imaging demonstrated mild diffusion restriction in the enhanced masses.

The patient subsequently underwent a bilateral simple mastectomy with immediate reconstruction. The resected breast specimens weighed 5275 g (right) and 6493 g (left) (Figure 1(b), (c)). Histological examination of the tissues revealed typical features of PASH, including stromal hyperplasia and slit-like spaces lined by endothelial-like spindle cells (Figure 1(d)). Immunohistochemical analysis showed positivity for CD34, vimentin, and smooth muscle actin and negativity for endothelial markers such as CD31 and von Willebrand factor.

The reporting of this study conforms to the CARE guidelines.^10^ Written informed consent was obtained from the patient to publish her anonymized data. Ethics committee approval was not required because of the retrospective nature of this case report.

## Discussion

PASH typically presents as a unilateral, solitary, and well-defined mass. In some cases, it may occur bilaterally, forming multiple masses.^11^ Rarely, PASH can present as diffuse lesions in both breasts, leading to significant breast enlargement as observed in our patient. Most specimens contain ≤50% PASH, supporting the view that PASH is usually an incidental finding.^12^ However, in our case, the PASH component accounted for >90% of the specimen, which is extremely rare. Additionally, a few cysts and fibroadenomas were identified within the specimen.

The etiology of PASH is believed to be strongly hormone-dependent^3,8,9^ because estrogen and progesterone receptors are present in most cases. A widely accepted hypothesis suggests that stromal proliferation in PASH results from an overreaction of myofibroblasts to hormonal stimulation.^1,13^ However, our patient’s estrogen and progesterone levels were within the normal range.

PASH is a benign breast condition characterized by a complex network of slit-like spaces lined by slender spindle cells on a background of stromal hyperplasia.^1^ These slit-like spaces may be mistaken for low-grade angiosarcoma. However, angiosarcoma can be differentiated based on its malignant cytologic features and positive immunohistochemical staining for endothelial markers, including CD31 and factor VIII-related antigen.^14–16^

Because PASH is usually diagnosed incidentally during histological examination of other benign or malignant lesions,^8,9^ surgical excision has traditionally been recommended to confirm the absence of occult malignancy and to prevent progression or recurrence.^2^ In recent years, the management of PASH has become more standardized. The American Society of Breast Surgeons does not recommend routine excision,^17^ although supportive data remain limited. Moreover, there is currently no statistical evidence of disease progression or reliable data regarding the most appropriate treatment for PASH.^18^ The clinical management of PASH remains a controversial issue. For rare diffuse cases causing significant breast enlargement, surgery may be considered; the recurrence rate for such cases ranges from 15% to 22%.^8^ In our case, the lesion rapidly enlarged within 2 years after surgery, leading to severe clinical symptoms. Our radiologist accurately diagnosed the condition preoperatively, and the clinician subsequently performed a mastectomy to prevent recurrence.
